# Psychometric Properties of and Measurement Invariance in the Questionnaire of Stereotypes Toward Older Adulthood in Health Care College Students and Health Professionals of Colombia: Psychometric Study

**DOI:** 10.2196/42340

**Published:** 2023-03-09

**Authors:** Marta Martín-Carbonell, Begoña Espejo, Greys Patricia Castro-Melo, Doris Sequeira-Daza, Irene Checa

**Affiliations:** 1 Universidad Cooperativa de Colombia Santa Marta Colombia; 2 Universitat de València Valencia Spain; 3 Universidad Central de Chile Santiago de Chile Chile

**Keywords:** psychometric properties, structural equation modeling, older adulthood, geriatric, gerontology, health care college students, health care professionals, questionnaire, stereotype, agism

## Abstract

**Background:**

In health professionals, negative stereotypes toward older adulthood have been associated with the difficulty in recognizing pathological processes and the refusal to care for older patients because of assuming that communication with them will be uncomfortable and frustrating. For these reasons, research on stereotypes in these groups has acquired growing importance. The usual strategy to identify and evaluate agist stereotypes is to use scales and questionnaires. Although multiple scales are currently used, in Latin America, the Questionnaire for the Evaluation of Negative Stereotypes Toward Older Adulthood (Cuestionario de Estereotipos Negativos sobre la Vejez [CENVE]), developed in Spain, is widely used but without evidence of construct validity in our context. In addition, although in the original version, a factorial structure of 3 factors was found, in later studies, a unifactorial structure was obtained.

**Objective:**

The objective is to study the construct validity of the CENVE in a sample of Colombian health personnel to clarify its factorial structure and concurrent validity. Likewise, the measurement invariance according to gender and age was studied.

**Methods:**

A nonprobabilistic sample of 877 Colombian health professionals and intern health students was obtained. The data were collected online using the LimeSurvey tool. To study the factor structure of the CENVE, 2 confirmatory factor analysis (CFA) models were carried out, one to test a single factor and the other to test the 3-related-factor structure. The factor measurement reliability was evaluated with the composite reliability index (CRI) and the average variance extracted (AVE). The measurement invariance was studied according to gender (men and women) and age (emerging adults, 18-29 years old, and adults, 30 years old or older). Using a structural equation model, the relationship between age and the latent CENVE total score was studied to obtain evidence of concurrent validity, since studies indicate that the younger the age, the greater the number of stereotypes.

**Results:**

The 1-factor structure was confirmed. The reliability results indicated that both indices show adequate values. Likewise, the existence of a strong invariance in measurement by gender and age group was verified. After contrasting the means of the groups, the results showed that men show more negative stereotypes toward old age than women. Likewise, emerging adults also showed more stereotypes than adults. We also verified that age is inversely related to the latent score of the questionnaire, such that the younger the age, the greater the stereotype. These results are in agreement with those obtained by other authors.

**Conclusions:**

The CENVE shows good construct and concurrent validity, as well as good reliability, and it can be used to assess stereotypes toward older adulthood in Colombian health professionals and health sciences college students. This will allow us to better understand the effect of stereotypes on agism.

## Introduction

The impact of negative stereotypes on the health of older people has been evidenced in several meta-analyses and recent multinational studies, which report the effects of age discrimination in multiple health domains, including longevity, quality of life, mental health, and cognitive impairment, among others [1-3]. The aging rate in Colombia has tripled in recent decades [4]. This demographic aging poses challenges to health systems, since older people are the main users of health services [4,5]. This situation makes it increasingly important for older people to be able to interact with workers free from negative stereotypes toward older adulthood.

Unfortunately, numerous studies report the existence of these stereotypes in health professionals, regardless of their specialties, in almost all the countries analyzed [6-10]. Manifestations of age discrimination are also evident in the education and training of professionals who care for the health of older adults [11]. In health professionals, negative stereotypes toward older adulthood have been associated with the difficulty in recognizing pathological processes [6] and the refusal to care for older patients because of assuming that communication with them will be uncomfortable and frustrating [10]. In countries with a lower level of development, age discrimination is expressed in a lower number of resources allocated for health care for the elderly [12]. Negative stereotypes also affect how other health professionals are perceived by other people, as well as how they perceive themselves. This situation leads to a decrease in job opportunities and an increase in job stress, especially among older professionals [1]. For these reasons, research on stereotypes in health professionals and health sciences college students has acquired growing importance, even impacting the political agendas of international and national organizations [2].

The existence of age discrimination in health care settings makes it necessary to target interventions at administrators, physicians, nurses, personal caregivers, and other associated health professionals, as well as trainee professionals [13]. Eradicating age discrimination from health care is not an easy task, especially the more indirect and subtle forms of discrimination, such as clinical decisions based on agist stereotypes [14]. It is recognized that interventions that are not designed on the basis of empirical evidence have the potential to do more harm than good [15]. Thus, identifying these stereotypes is an essential prerequisite for interventions and policies aimed at eradicating age discrimination in health care.

Stereotypes are a priori, biased, and unproven ideas that assign certain attributes to people on the assumption that they belong to a group with homogeneous characteristics [16,17]. The study of stereotypes toward older people is related to the concept of agism [18,19], which refers to prejudice toward people just because they are of a certain age [20]. Age discrimination is similar to other known forms of discrimination, such as discrimination based on gender or ethnicity [5]. Like these, at the individual level, it has a cognitive component (stereotype), an affective component (prejudice), and a behavioral component (discriminatory behaviors) [2].

In the literature, it is possible to find a large number of texts that use the term “agism” to refer to negative stereotypes toward older people [12,14]. Therefore, in this paper, we use them as synonyms, as is the usual practice.

Multiple theories have attempted to explain the occurrence of agism. Ayalon and Tesch-Römer [12] classify these theories at 3 levels of agism: micro, meso, and macro levels. The micro level is concerned with the individual (thoughts, emotions, actions), the meso level is concerned with groups (eg, age, gender) and other social entities (eg, in the domain of work or health care services), and the macro level relates to cultural or societal values (eg, political regulations). At an individual level, a sociological and psychological explanation of agism can be found in terror management theory. According to this theory, agism is seen as an unconscious defense against death anxiety, which might arise because of the encounter with the old age group. However, some studies indicate that this mechanism is relevant mostly among young and middle-aged groups, and it becomes less relevant among the old-old age group. This suggests a gradual reduction in death anxiety in this age group and a greater acceptance of the inevitability of death [12].

Evidence has also been found that young people perceive older adults as bad tempered, cheerless, isolated, poor, senescent, unhealthy, unable to learn, and useless or unable to work efficaciously [21,22]. One explanation is that in most modern Western societies, there is a clear segregation between the young and the old based on preplanned life scripts, which include education, family creation, work, and retirement [12]. Additionally, intergenerational conflict theory proposes 3 bases for intergenerational conflict, which are exacerbated by the expectations that younger generations have of older generations and age-appropriate symbolic identity maintenance [23].

It is suggested that stereotypes toward old age can be ambivalent, since they can contain some positive attributes, such as warmth, prestige, or wisdom [2], but they are mainly characterized by being negative evaluations related to physical or mental illnesses, disability, lack of interests and vital motivations, social disengagement, inactivity, or uselessness [24-26]. The stereotype content model [27] proposes that stereotypes toward older adults involve perceptions of older adults as warm (a positive trait) but incompetent (a negative trait). This combination of high warmth and low competence leads to paternalistic prejudice, which is associated with several negative outcomes [28].

Studies in Latin America and Colombia [29,30] have shown that the view of older adulthood prevails as a stage of life with little participation in society, characterized by having multiple diseases and a considerable increase in disability. Unfortunately, we have found few studies focused on the stereotypes of health personnel about older people in Colombia, but they agree that this stereotyped negative vision prevails [31-33].

The usual strategy to identify and evaluate agist stereotypes is to use scales and questionnaires. Although multiple scales are currently used, evidence on the psychometric properties of these scales is scarce [34]. In their systematic review, studies also warn that the scales only assess the cognitive component (stereotypes) but not the affective (prejudice) and behavioral (discrimination) components. Among the questionnaires that are most mentioned in the literature are the Scale of Attitudes towards the Elderly [35], the Semantic Differential Scale of Aging [36], the Facts on Aging Questionnaire [37], and the Fraboni Scale of Ageism [38]. However, as far as we know, none of these instruments have been validated in the Colombian population.

In the Latin American sphere, and in Colombia in particular, the most widely used instrument is the Questionnaire for the Evaluation of Negative Stereotypes Toward Older Adulthood (Cuestionario de Estereotipos Negativos sobre la Vejez [CENVE]) [30,32,39,40]. The CENVE was developed in Spain, more than 15 years ago, by a team of psychologists from the University of Malaga [18]. After performing a principal component analysis, a structure of 3 related factors was found: one factor called health, another factor called motivational-social, and the third factor called character-personality.

The CENVE has been used in numerous studies to investigate stereotypes in health sciences professionals and students from various Spanish-speaking countries, such as Spain [38,39], Peru [40], Uruguay [33], El Salvador [41], Costa Rica [42], Argentina [43], Chile [44,45], Mexico [8,46], and Colombia [35,36]. It has been adapted in Portugal, too [37].

However, subsequent studies conducted to analyze the construct validity of the CENVE do not support the 3D structure. In a study carried out in Spain, the existence of these 3 factors could not be confirmed, but good indicators for a 1D structure were obtained [19]. A similar result was reported in Portugal [41]. However, in Chile, a revised version was also recently proposed that modifies the form and order of the questions and the wording of some items, and a new item was included that evaluates the stereotype that older people isolate themselves from their environment [42]. Regarding the construct validity of this version, it was found that it also shows a 1D structure.

As can be seen from these findings, few studies have examined the validity of the CENVE and none have been reported in Colombia. According to the International Test Commission [43], the most important thing is to obtain evidence that a construct is really being measured, in this case 1D, and verify that there is an underlying dimension to all the items in accordance with the previous theory.

In view of these few studies, it can be confirmed that there is still not enough evidence of the factorial structure of the CENVE in Spanish-speaking countries outside of Spain, and as we have already pointed out, we are not aware of any study in Colombia. In addition, the few psychometric studies carried out do not support the structure of 3 related factors. For this reason, it is important to investigate the psychometric properties of this measurement instrument, since it is being used without evidence of validity in our context. Therefore, in this report, we studied the construct validity of the CENVE in a sample of Colombian health personnel to clarify its factorial structure and concurrent validity. The relevance of investigating invariance is underlined by previous findings on the relationship of these variables with agist stereotypes [6,7,9,24,26,30,39,44].

## Methods

### Participants

A nonprobabilistic sample of 877 Colombian health care professionals and intern health students residing in various regions of Colombia was obtained, although people from the Caribbean region predominated (n=847, 96.6%). The average age of the sample was 28.49 (SD 8.66) years, ranging from 18 to 75 years. Most of the participants were women (n=512, 58.4%), 331 (37.7%) were men, 10 (1.1%) identified themselves with another gender, and 24 (2.7%) preferred not to answer. Of the total participants, 493 (56.2%) were final-year students with internships, 375 (42.8%) were professionals, and 9 (1%) did not answer this question. Most of the students were medicine (n=368, 42%) and nursing (n=270, 30.8%) students, followed by psychology (n=146, 16.6%), social work (n=21, 2.4%), physiotherapy (n=18, 2%), and occupational therapy (n=4, 0.4%) students. The rest were studying for other degrees (n=50, 5.7%). Regarding health professions, the majority were professionals in nursing (n=124, 38.6%) and medicine (n=105, 32.3%), followed by graduates in psychology (n=44, 15.3%), social work (n=20, 7.3%), physiotherapy (n=14, 5.2%), and occupational therapy (n=4, 1.3%). The rest were professionals with other qualifications (n=68, 24.7%). (There were people with more than 1 degree, which is why the percentage adds up to more than 100.) Regarding years of professional experience, the minimum value was less than 1 year of experience (n=111, 12.6%) and the maximum value was up to 45 years of experience. The mean was 7.14 (SD 7.03) years of experience.

### Instrument

To evaluate negative aging stereotypes, we used the Spanish version of the CENVE [18], since after the qualitative study carried out by experts, it was concluded that the items were worded appropriately for the Colombian population. This questionnaire comprises 15 items that are answered with a Likert-type scale ranging from 1 (strongly disagree) to 4 (strongly agree), and participants are asked to think of people 65 years of age or older when responding to the items. The original version showed a structure of 3 factors comprising 5 items each. The stereotypes toward older adulthood evaluated in each factor were referred to as health, motivation, and personality. The higher the score on a factor, the higher the level of negative aging stereotypes.

### Procedure

The first step was to study the content validity of the original CENVE, considering the age of the instrument and the results of Rosell et al [42] in Chile. For this, we formed an evaluation group comprising 9 Colombian judges (3 doctors, 3 psychologists, and 3 nurses, 33.3% each) with different levels of experience in caring for the elderly and in the use of attitude evaluation instruments. They had to complete a structured interview to evaluate the items according to 4 criteria: clarity of the items, sensitivity to variations in the phenomenon being measured, justification for the presence of each of the items, and whether they considered it essential or important (if it had to be included). This assessment was conducted using a scale from 1 to 5 (the higher the score, the better the assessment). The item was considered adequate when at least 70% of the experts gave a score greater than 3 in all the criteria evaluated. The results of the evaluations were discussed in the research team, reaching a consensus that all the items exceeded the filter, so it was appropriate to use the original scale.

Data were collected online between August 1, 2021, and May 20, 2022, using the LimeSurvey platform installed on the university's servers. The form included questions to obtain sociodemographic and professional information. The link to it was sent via email and distributed on social networks, following the snowball process. The study was briefly explained before starting the survey, and the participants had to provide informed consent to begin responding.

### Statistical Analysis

To obtain evidence of validity based on the internal structure of the CENVE, 2 confirmatory factor analysis (CFA) models were calculated, one to test a single factor and the other to test the 3-related-factor structure. The weighted least squares means and variances (WLSMV) adjusted estimator was used due to the ordinal nature of the response scale. To test the fit of the models, the usual fit indices were used. The reference values were 0.90 for the comparative fit index (CFI) and a maximum cut-off of 0.08 for the root-mean-square error of approximation (RMSEA) and for the standard root-mean-square residual (SRMR) to consider them indicative of a good-fit model [45-47]. The factor measurement reliability was evaluated with the composite reliability index (CRI) [48,49], which is identical to the ω coefficient [50] because the standardized factor loadings have been used. Next, the average variance extracted (AVE) was calculated to estimate the proportion of variance explained by each factor. Values equal to or greater than 0.70 for the CRI and values equal to or greater than 0.50 for the AVE are considered good [48]. For the model that best fit the data, the corrected item-total polyserial correlations for the items were calculated as indicators of corrected homogeneity indices for items with ordinal response scales [47].

Likewise, the measurement invariance according to gender and age was studied for the best model, evaluated by calculating 3 nested invariance models that impose successive restrictions: configural, metric, and scalar. To study invariance by gender, only 2 groups were considered: men and women. To study the measurement invariance by age, 2 groups were created: emerging adults (from 18 to 29 years old) and adults (30 years old or older) [51].

To assess the degree of invariance among the models, the following cut-off points in the increase in the indices were considered: a change of 0.010 or greater in the CFI, along with a change of 0.015 or greater in the RMSEA, or a change of 0.030 or greater in the SRMR would indicate that there is no invariance [52].

In addition, to obtain evidence of concurrent validity, the correlation between age and the latent factor (or factors) of the best model obtained in CFA was estimated. Since we previously tested the 1-factor model and the 3-related-factor model, if the model that best fit the data was the 3-factor model, a model in which age correlated with each latent factor would be tested. If the model that best fit the data was a 1-factor model, a model in which age correlated with that factor would be tested. The expected result was that the younger the age, the higher the level of stereotypes. Finally, descriptive statistics and norms for the total score (percentile rankings) were calculated.

CFA, corrected item-total polyserial correlations, and measurement invariance analyses were carried out with Mplus 8.8 [53], and to describe sociodemographic variables and statistics for the items of the CENVE and for the scale, IBM SPSS Statistics version 28 was used.

### Ethical Considerations

The survey was completely anonymous and voluntary, and the participants did not receive financial or any other kind of compensation. The LimeSurve platform used to collect the data is installed on the university's servers, with which the storage and custody of the data remain in the hands of the university and not third parties. The study was conducted in compliance with Colombian legislation (Ley Orgánica 3/2018, December 5) and the code of ethics for research involving human subjects, as outlined by the Cooperative University of Colombia [54]. All participants provided informed consent to begin responding.

## Results

### Evidence of Validity Based on the Internal Structure, and Reliability

Two CFA models were tested to confirm the structure of the CENVE in a Colombian sample. Although some fit indices could suggest that the model fit was adequate for the 3D model, *χ*^2^_87_=794.8 (*P*<.001), CFI=0.930, RMSEA=0.096 (90% CI 0.090-0.103), and SRMR=0.046, the latent variable covariance matrix was not positive definite. This may be due to a negative variance/residual variance for a latent variable, a correlation greater than or equal to 1 between 2 latent variables or a linear dependency among more than 2 latent variables. Furthermore, the correlations between the factors were inadequate, since 1 of them showed a value greater than 1 (1.040) and the other 2 showed values close to 1 (0.963 and 0.943).

However, the 1-related-factor model showed a higher *χ*^2^ and similar fit indices, being a parsimonious solution and showing a good fit except for the RMSEA: *χ*^2^_90_=834.1 (*P*<.001), CFI=0.926, RMSEA=0.097 (90% CI 0.091-0.103), and SRMR=0.048. Although the RMSEA and CFI values are inconsistent in this case, some authors say that it can happen at times because the CFI and RMSEA are calculated differently, so they are not comparable qualitative assessments. When the RMSEA and CFI offer different assessments of the fit of the model, some authors argue that this does not mean that the model is poorly specified or that there is a problem with the data. These indices may differ in their interpretation because they assess the fit of the model from different perspectives [55]. The RMSEA is a nonstandardized fit index that can be interpreted only using arbitrary cut-offs. However, the CFI is a relative measure of improvement in fit [56]. Regarding the SRMR and RMSEA, other authors indicate that the SRMR, being a standardized fit index, shows a higher power to reject models that present poor fit to the data with ordinal responses (as in this case) regardless of the number of parameters to be estimated and the sample size [56]. Therefore, the fit of the model can be evaluated using the SRMR and CFI. For these reasons, we consider that the 1-factor model for the CENVE showed a good fit to the data in this sample.

Additionally, all factor loadings were statistically significant (*P*<.001) and ranged from 0.65 to 0.74. Likewise, CRI=0.93 and AVE=0.70 were good values. Corrected item-total corrected polyserial correlations (Table 1) also showed adequate values, ranging from 0.61 to 0.71 (SE 0.014-0.020).

**Table 1:** Statistics and corrected item-total polyserial correlations for the 1-factor model of the items of the CENVE^a^.

Item	Mean (SD)	Skewness	Kurtosis	Item-total corrected polyserial correlation	SE for the item-total corrected polyserial correlation
Item 1	3.06 (0.86)	–0.77	0.10	0.61	0.017
Item 2	2.78 (0.79)	–0.51	0.02	0.63	0.018
Item 3	2.87 (0.88)	–0.39	–0.57	0.61	0.021
Item 4	2.67 (0.87)	–0.31	–0.55	0.65	0.016
Item 5	2.60 (0.97)	–0.09	–0.98	0.64	0.019
Item 6	2.83 (0.82)	–0.48	–0.16	0.69	0.016
Item 7	2.91 (0.76)	–0.52	0.18	0.65	0.019
Item 8	2.75 (0.87)	–0.26	–0.62	0.68	0.018
Item 9	3.12 (0.90)	–0.91	0.15	0.59	0.021
Item 10	2.77 (0.74)	–0.54	0.27	0.70	0.017
Item 11	2.68 (0.81)	–0.18	–0.45	0.64	0.017
Item 12	2.78 (0.86)	–0.44	–0.37	0.65	0.017
Item 13	2.99 (0.82)	–0.50	–0.28	0.64	0.020
Item 14	2.72 (0.87)	–0.40	–0.43	0.69	0.015
Item 15	2.90 (0.79)	–0.51	0.04	0.64	0.019

^a^CENVE: Cuestionario de Estereotipos Negativos sobre la Vejez.

### Measurement Invariance

The results for the measurement invariance model by gender and age (Table 2) showed a good fit of the 1-factor model in both gender groups, especially for men, and in both age groups, especially for adults. Again, the same happened with the value of the RMSEA in these models, as well as in the models used to test the measurement invariance. Therefore, we can consider that the 1-factor model fits the 4 groups.

Observing the changes in the CFI and RMSEA values, we can consider that the results showed scalar invariance by gender and age group. Therefore, the estimated latent means can be compared. After fixing the latent mean values to 0 for women, men showed a statistically higher mean in stereotypes toward older adulthood (b=0.133, *z*=2.77, *P*=.006). Regarding age, after fixing the latent mean values to 0 for emerging adults, the emerging adults showed a statistically higher mean in stereotypes toward older adulthood (b=–0.434, *z*=–3.31, *P*=.001).

**Table 2:** Measurement invariance by gender and age models and goodness-of-fit indices.

Model			Indices						Change in indices		
		*χ*² (*df*)		Δ*χ*²^a^ (Δ*df*^b^)	CFI^c^	RMSEA^d^	SRMR^e^	ΔCFI		ΔRMSEA	ΔSRMR
**Gender**											
	Men (reference)	367.26^f^ (90)		N/A^g^	0.923	0.096	0.058	N/A		N/A	N/A
	Women	615.15^f^ (90)		N/A	0.919	0.107	0.054	N/A		N/A	N/A
**Nested invariance models for gender**											
	Configural	958.78^f^ (180)		N/A	0.923	0.101	0.055	N/A		N/A	N/A
	Metric	944.82^f^ (194)		13.96 (16)	0.926	0.096	0.056	0.003		–0.005	0.001
	Scalar	1067.73^f^ (223)		122.91 (29)	0.917	0.095	0.059	0.009		–0.001	0.003
**Age**											
	Emerging adults (reference)	712.57^f^ (90)		N/A	0.904	0.109	0.054	N/A		N/A	N/A
	Adults	332.57^f^ (90)		N/A	0.929	0.096	0.062	N/A		N/A	N/A
**Nested invariance models for age**											
	Configural	976.97^f^ (180)		N/A	0.922	0.100	0.057	N/A		N/A	N/A
	Metric	943.08^f^ (194)		33.89 (16)	0.927	0.094	0.057	0.005		–0.006	0
	Scalar	974.53^f^ (223)		31.45 (29)	0.927	0.088	0.059	0		–0.006	0.002

^a^Δ*χ*²: chi-square change.

^b^Δ*df*: degrees-of-freedom change.

^c^CFI: comparative fit index.

^d^RMSEA: root-mean-square error of approximation.

^e^SRMR: standardized root-mean-square residual.

^f^*P*<.001.

^g^N/A: not applicable.

### Evidence of Validity Based on the Relationship With Other Variables

**Figure 1:**
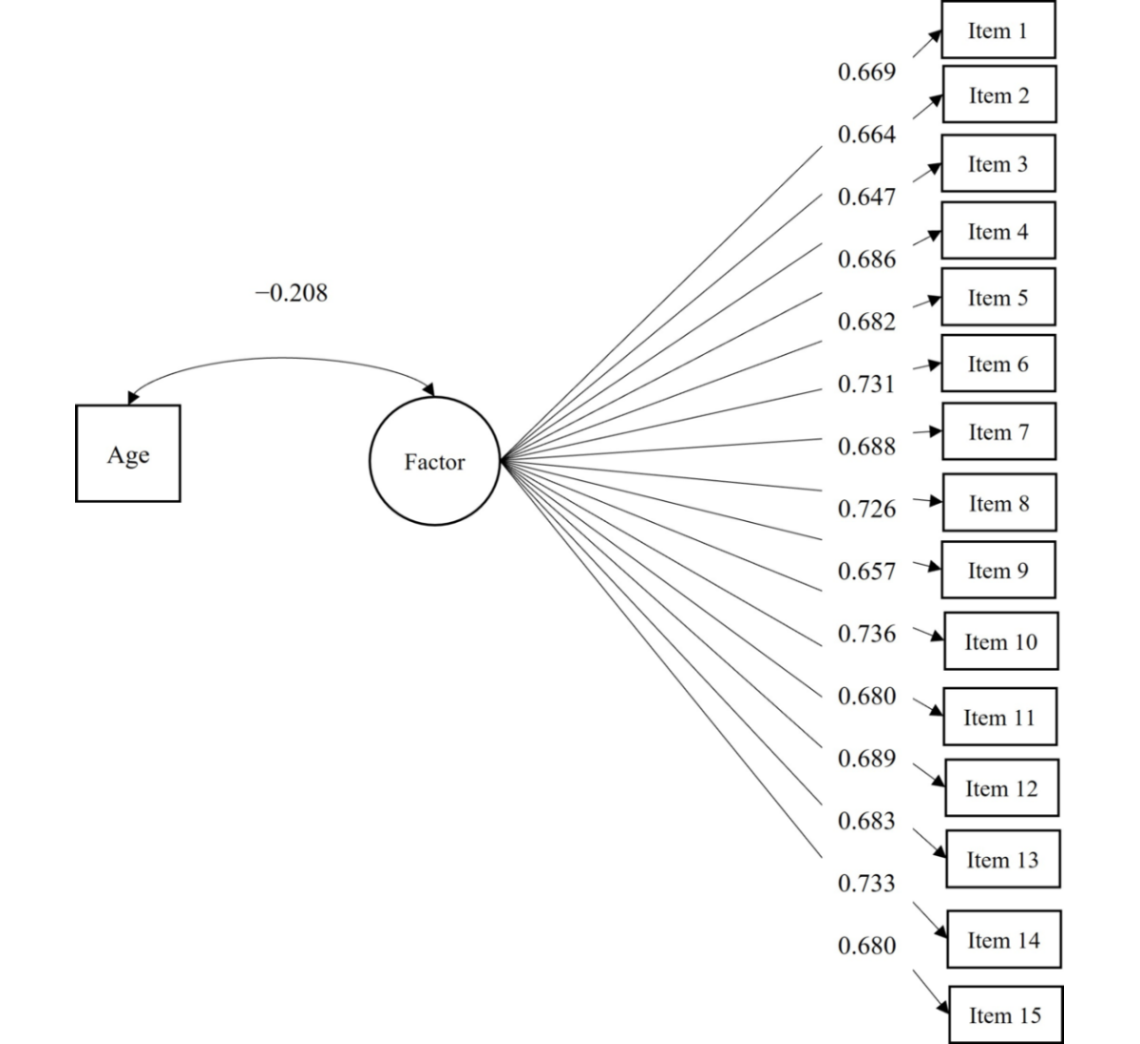
Validity model for the 1-factor structure of the CENVE. All factor loadings and the correlation between age and the factor were statistically significant (*P*<.001). CENVE: Cuestionario de Estereotipos Negativos sobre la Vejez.

Results for the estimated concurrent validity model with the 1-factor structure (Figure 1) indicated a good fit of the model: *χ*^2^_104_=840.97 (*P*<.001), CFI=0.930, RMSEA=0.090 (90% CI 0.084-0.096), and SRMR=0.047. Again, the same happened with the value of the RMSEA, but looking at the other fit indices, we can consider that the model fits well. The correlation between the age of the participants and the latent factor of the CENVE was negative and statistically significant, as expected, which means that the older the age, the fewer the stereotypes toward older adulthood.

### Normative Data in This Sample

In Table 3 are shown the descriptive statistics for the total CENVE score, as well as the percentiles obtained in the sample of this study.

**Table 3:** Descriptive statistics and normative data (percentile scores) for the total score of the CENVE^a^.

Variable		Value
N		877
Mean (SD)		42.43 (8.39)
Median		44
Mode		45
Skewness		–0.58
Kurtosis		0.78
Minimum		15
Maximum		60
**Percentiles**		
	5	28
	10	32
	15	34
	20	36
	25	38
	30	39
	35	40
	40	41
	45	42
	50	44
	55	45
	60	45
	65	45
	70	47
	75	48
	80	49
	85	51
	90	52.2
	95	54

^a^CENVE: Cuestionario de Estereotipos Negativos sobre la Vejez.

## Discussion

### Principal Findings

The objective of this study was to study the psychometric properties of the CENVE in a general Colombian sample of health professionals and health sciences college students. This study fills the gap in the limited information about instruments to assess stereotypes toward older adulthood in our context. The results indicate that the 1-factor model does show a good fit, while the model of 3 related factors described by the authors of the instrument does not show a good fit. This result coincides with those obtained by other authors in which, after carrying out a CFA for each model (1 and 3 factors), the 1-factor model is the one that best fits the data [19,41,42]. It should be noted that in the original study by Blanca-Mena et al [18], the strategy to obtain the factorial structure was not the most appropriate for the nature of the Likert scales. In addition, principal component analysis allows us to summarize the observed scores in a large set of observed variables but not the number and composition of the common factors (latent variables) necessary to explain the common variance of the set of items analyzed [57]. However, the identification of 3 dimensions proposed for the rating by Blanca-Mena et al [18] is based only on these empirical data, without further theoretical support.

Negative stereotypes toward old age are usually considered the cognitive dimension of agism. Our study indicates that for our environment, it should be considered that the CENVE offers a single global measure, with the highest scores corresponding to people with more negative stereotypes. In this negative and harmful vision of old age, the CENVE includes elements of evident hostility (eg, older people are easily irritated and grouchy) and others that can be considered benevolent (eg, older people are often like children) but that show evidence of its negative effect on the health and quality of life of the elderly [58]. The fact that age stereotypes are a reason for age discrimination is firmly established in research [1,2,5,12,14], and having an instrument that allows them to be identified constitutes the essential first step for any intervention aimed at correcting age discrimination and its adverse effects on the health of the elderly. In this sense, our results warn about the predominance of negative stereotypes in the investigated sample.

However, in our study, we previously verified that the original questionnaire can be used in our environment since it is well understood by Colombian professionals and students of health sciences. The reliability results of the questionnaire indicate that both the CRI and the AVE show adequate values. Likewise, the values of the corrected homogeneity indices show good values.

To the best of our knowledge, this is the first study that reports on this evidence of the construct validity of the CENVE in Colombia. Additionally, the invariance in measurement by gender and age group was studied, which had not been studied until now, except in the modified version of this questionnaire [42]. The results indicate that there is scalar measurement invariance by gender and age group. Therefore, the CENVE can be used to compare groups of people of different ages (emerging adults and adults) and genders (male and female), with the certainty that the same thing is being evaluated in the same way. This will allow us to increase our understanding of the effect of these variables on agism.

After comparing the means for the groups in this sample, it is found that men and emerging adults show higher scores on the scale of stereotypes toward older adulthood. This significant difference regarding gender was also found in a study that was carried out with the modified CENVE scale [42], and it is consistent with the various studies that refer to more agist stereotypes in men than in women [5,8,12,16,21]. Likewise, and in this sense, we also found evidence of concurrent validity by verifying how age is inversely related to the CENVE latent score, since the model shows that younger people show higher levels of stereotypes. These results are in agreement with those obtained by other authors in different general samples [6,8,9,19,59,60].

### Limitations

This study had a few limitations. Maybe the most important is that the sample was not representative of Colombian health care professionals or health care sciences college students, since most of the participants were from the Caribbean region (about 70%). We had to resort to sampling by availability, which limits the generalization of the results, especially considering the cultural diversity of Colombia.

Another limitation is that there was no information derived from other sources that provided us with evidence of convergent validity, which could have been obtained if other instruments on stereotypes toward older adulthood had been available. Information about the stability of the CENVE scores and their sensitivity to detect changes derived from specific interventions was also not offered, which should be the subject of future studies. For these reasons, we recommend that the normative values provided be used with caution and for research purposes only.

Despite these limitations, and based on the strength of the results found, it can be concluded that the CENVE can be used for the evaluation of stereotypes toward the elderly, both in health professionals and in students of Colombian health sciences. However, due precautions derived from the aforementioned considerations must be kept in mind. This will allow us to increase our understanding of the effect of these variables on agism and to detect changes derived from specific interventions in the future.

In future studies, it will be interesting to analyze the measurement invariance between young people and older people, since young people predominate in this sample and the groups used to study invariance by age are emerging adults and adults. Furthermore, it would also be convenient to study the functioning of the response scale, since some research suggests that there may be problems with the order of the Likert-type response alternatives [61-64] as they may not be ordered as expected according to the response system. This would lead to a reliability problem of the questionnaire.

### Conclusion

We can conclude that the questionnaire shows good construct and concurrent validity, as well as good reliability. So, it can be used to assess stereotypes toward older adulthood in Colombian health professionals and health sciences college students. As some studies show, negative stereotypes toward older adulthood have been associated with difficulty in recognizing pathological processes and the refusal to care for older patients because of assuming that communication with them will be uncomfortable and frustrating.

Therefore, knowing the stereotypes toward older adulthood that health professionals and people who are conducting this type of university studies show will allow us to increase our understanding of the effect of these variables on agism, since the results obtained it will support interventions to correct agist stereotypes, actions aimed at modifying these beliefs, in health professionals and college students in Colombia. These actions will influence better treatment of older patients.

## Abbreviations

AVE: average variance extracted
CENVE: Cuestionario de Estereotipos Negativos sobre la Vejez (Questionnaire for the Evaluation of Negative Stereotypes Toward Older Adulthood)
CFA: confirmatory factor analysis
CFI: comparative fit index
CRI: composite reliability index
RMSEA: root-mean-square error of approximation
SRMR: standardized root-mean-square residual
WLSMV: weighted least squares means and variances

## Data Availability

The data sets generated and analyzed during the study are not publicly available due to their proprietary nature but are available from the corresponding author upon reasonable request.
